# Editorial: The interplay between GABA and glutamate in systems physiology and pathophysiology

**DOI:** 10.3389/fphar.2026.1831636

**Published:** 2026-04-10

**Authors:** Claudio Rivera, Stephen J. Lewis

**Affiliations:** 1 INMED, Aix-Marseille Université, Marseille, France; 2 Neuroscience Center, HiLife, University of Helsinki, Helsinki, Finland; 3 Department of Pediatrics, Department of Pharmacology School of Medicine, Case Western Reserve University, Cleveland, OH, United States

**Keywords:** excitation-inhibition balance, GABAergic signaling, glutamatergic transmission, neuropharmacology, synaptic plasticity

Gamma-aminobutyric acid (GABA) and glutamate form the principal inhibitory and excitatory signaling systems of the nervous system. Their interplay is not a static opposition, but a dynamic and context-dependent relationship that shapes neural computation across scales—from receptor activation and intracellular chloride regulation to synaptic plasticity, circuit function, behavior, and disease. This Research Topic brings together clinical, translational, and experimental studies that illustrate how disturbances in this balance contribute to disorders as diverse as schizophrenia, insomnia, epilepsy, neurodegeneration, obesity, and drug-induced neurological toxicity. Taken together, these contributions underscore a central concept in modern neuropharmacology: therapeutic modulation of GABAergic and glutamatergic systems must be understood at the level of integrated physiology, not simply as isolated receptor pharmacology.

A first major theme emerging from this Research Topic is that inhibitory signaling cannot be reduced to “less activity,” nor excitatory signaling to “more activity.” Rather, the functional meaning of GABAergic modulation depends on cellular identity, subcellular localization, developmental state, chloride homeostasis, and the architecture of the surrounding network. This is particularly clear in the study GABAB receptors negatively modulate excitatory plasticity at the mossy fiber synapse onto parvalbumin-expressing basket and axo-axonic cells in the dentate gyrus, which shows that GABA_B_ receptors directly constrain long-term potentiation at excitatory inputs onto parvalbumin interneurons. These findings are important because they shift attention from principal cells alone to inhibitory neurons as active substrates of synaptic plasticity. In doing so, they highlight how GABAergic mechanisms can indirectly regulate glutamatergic throughput and network excitability by shaping the plasticity of inhibitory microcircuits themselves.

A related systems-level perspective is developed in “Altered GABAergic signaling and chloride homeostasis in eye movement circuits during late neurodevelopment: implications for Alzheimer’s disease therapy” (Porcher et al.). This review expands the classical excitation/inhibition framework by emphasizing the importance of intracellular chloride regulation, especially through KCC2, in determining whether GABA remains functionally inhibitory. By linking altered GABAergic signaling and chloride homeostasis to oculomotor dysfunction in Alzheimer’s disease, the article also argues that subtle physiological outputs such as eye movements may provide sensitive windows into circuit instability. More broadly, it reminds us that pathophysiological changes in GABA–glutamate interactions may be detectable long before overt neurodegeneration becomes clinically obvious.

Another strength of this Research Topic is the way it bridges molecular and circuit mechanisms with human disease. In “Serum levels of S100B in patients with chronic schizophrenia during treatment augmentation with sarcosine: results of the double-blind, randomized, placebo-controlled PULSAR study” (Pawlak et al.), sarcosine augmentation improved negative and general psychopathology symptoms without significantly altering serum S100B concentrations. This negative result is scientifically valuable. It refines the relationship between glutamatergic modulation, glial markers, and clinical response, suggesting that symptomatic improvement may occur independently of measurable changes in this peripheral index of glial pathology. At the same time, the exploratory association between S100B dynamics and affective symptoms points to the continued need for multidimensional biomarkers that capture not only neurotransmission, but also glial and inflammatory contributions to psychiatric disease. Schizophrenia, as this study illustrates, cannot be understood through a neuron-centric lens alone.

The importance of non-neuronal and non-canonical modulators is also highlighted by “Kininogen enhances seizure susceptibility in mice possibly through bradykinin-induced modulation of calcium transients in glutamatergic and GABAergic neurons” (Ghosh et al.). Here, seizure susceptibility is increased in parallel with enhanced activity in glutamatergic neurons and reduced activity in parvalbumin-positive inhibitory neurons. This is an elegant example of how inflammatory or vascular-linked mediators can shift the excitation/inhibition balance by acting on both sides of the circuit equation. Epileptogenesis is therefore not merely a disorder of excessive excitation or failed inhibition, but a systems disorder in which immune-related signaling molecules reshape the interaction between the two.

Sleep regulation offers yet another angle on this problem. In “Insomnia treatment based on the regulation of the GABAergic system: traditional Chinese medicine perspectives and therapeutic approaches” (Cheng et al.), GABAergic signaling is presented as a central therapeutic target, but within a broader framework that includes the hypothalamic–pituitary–adrenal axis, neuroinflammation, circadian regulation, and neurotrophic support. This integrative approach is valuable because it resists overly reductionist interpretations of insomnia pathophysiology. GABA–glutamate balance does not operate in isolation; it is embedded within endocrine, immune, and circadian systems that determine overall brain state. The review also illustrates how multi-target treatment frameworks may offer conceptual advantages for disorders in which network dysregulation arises from several converging biological disturbances.

The translational reach of this Research Topic extends beyond the brain as conventionally defined. “Synergistic effects of peripheral GABA and GABA-transaminase inhibitory drugs on food intake control and weight loss in high-fat diet-induced obese mice” (Nagao et al.) shows that manipulation of GABA availability can influence feeding and body weight, emphasizing that GABAergic pharmacology also intersects with metabolic regulation. This broadens the physiological territory of GABA–glutamate research and suggests that future neuropharmacology will increasingly need to consider brain–body interactions when evaluating therapeutic mechanisms.

At the same time, therapeutic promise must be weighed against safety. “Neurological adverse events associated with baclofen: a pharmacovigilance study based on FDA adverse event reporting system” (Zhao et al.) provides an important reminder that drugs targeting inhibitory signaling can produce substantial neurological adverse events in real-world use. Baclofen remains a clinically valuable GABA_B_ receptor agonist, but its population-level safety profile emphasizes the need for individualized dosing, careful monitoring, and more precise strategies for modulating inhibitory tone. This is especially relevant as the field moves toward interventions targeting receptors, transporters, metabolic enzymes, and neuromodulatory pathways that influence GABA–glutamate balance.

Overall, the articles in this Research Topic converge on a common message: the interplay between GABA and glutamate is best understood as a systems property emerging from interactions among neurons, glia, synapses, ion transporters, modulatory peptides, metabolic pathways, and whole-organism physiology. Disease states do not simply “increase excitation” or “reduce inhibition”; they alter the rules by which these two signaling systems communicate across time and scale. The challenge for pharmacology is therefore not merely to push the balance in one direction, but to restore context-appropriate regulation.

By integrating studies ranging from synaptic plasticity and chloride homeostasis to clinical trials, pharmacovigilance, sleep medicine, epilepsy, obesity, and neurodegeneration, this Research Topic highlights both the complexity and the therapeutic potential of targeting GABA–glutamate interactions. It is precisely at this intersection—between mechanism and system, physiology and pathophysiology—that the next-generation of neuropharmacological advances is likely to emerge.

